# The complete mitochondrial genomes of *Notropis chlorocephalus* and *Notropis chiliticus*

**DOI:** 10.1080/23802359.2025.2457449

**Published:** 2025-01-23

**Authors:** Zachariah D. Alley, Kayla M. Fast, Michael W. Sandel

**Affiliations:** a Protected Species Practice, EDGE Engineering and Science, LLC, Houston, TX, USA; b Department of Wildlife, Fisheries and Aquaculture, Mississippi State University, Mississippi State, Mississippi, USA; c Forest and Wildlife Research Center, Mississippi State University, Mississippi State, Mississippi, USA

**Keywords:** Leuciscidae, genome, mitochondria, minnow

## Abstract

We present a novel mitogenome assembly of the Redlip Shiner, *Notropis chiliticus*, and assemblies for the Greenhead Shiner, *N. chlorocephalus* (Cypriniformes: Leuciscidae). Both are charismatic minnows in the taxonomic group *Hydrophlox* and are endemic to the eastern United States. The *N. chiliticus* genome contains 16,711bp and *N. chlorocephalus* 16,706bp each comprising a total of 13 protein coding genes, 22 tRNAs, two rRNAs, and a control region. Sequence order and genome composition are similar to other *Notropis.* We provide evidence supporting a monophyletic *Hydrophlox*, while other phylogenetic relationships within *Notropis* support convoluted taxonomic revisions throughout the history of the genus.

## Introduction

The Greenhead Shiner, *Notropis chlorocephalus* (Cope 1870), and the Redlip Shiner, *N. chiliticus* (Cope 1870), are members of the recently elevated genus *Hydrophlox* (Gilbert and Jenkins 1978) (Cypriniformes, Leuciscidae; Stout et al. 2022; Page et al. 2023). This is a group of minnows known for bright nuptial coloration, communal spawning behavior, and obligate association with nests constructed by the genus *Nocomis* (Etnier and Starnes 1993). *Notropis chlorocephalus* is a polymorphic species known to occur in the Catawba River watershed of the Santee Basin and the upper Lynches River system of North and South Carolina (Wood and Mayden 1992; Rohde et al. 2009; Cashner et al. 2011). *Notropis chiliticus* is native to the Roanoke and Pee Dee drainages of Virginia, North Carolina, and South Carolina with assumed introductions in the Cape Fear, Santee, and New River drainages (Page and Burr 1991; Rohde et al. 2009). Both species are listed by the International Union for the Conservation of Nature (IUCN) as ‘Least Concern’.

Nonindigenous occurrences of *Notropis* shiners have increased in frequency throughout the southeastern United States (Etnier and Starnes 1993; Scott et al. 2009). *Notropis* shiners are abundant and conspicuous in suitable habitat; therefore, the likelihood of capture by recreational anglers in search of bait is high. This supports the widely propounded theory of anthropogenic introductions *via* release of bait as an explanation for non-native occurrences (Ludwig and Leitch 1996; Fuller et al. 2000; Scott et al. 2009; Kilian et al. 2012; Mooney et al. 2021). While there are few known negative impacts of *Notropis* invasion, non-native introductions can produce a cascade of negative effects (Taniguchi et al. 2002). Early detection of introduced species is a considerable challenge, yet it can be a critical component of mitigating ecological harm (Berec et al. 2015). While physical specimens are crucial for confirming non-native species occurrences, early detection can be achieved by other means. Environmental DNA (eDNA) can serve as a means of identifying potential invasions by non-native organisms before they reach abundances detectable in standardized surveys (Mehta et al. 2007). Here, we present the complete mitochondrial genomes of *N. chlorocephalus* and *N. chiliticus* (novel), with the aim of strengthening means by which occurrences of *Notropis* shiners can be detected, as well as increasing the body of data available for phylogenomic analysis.

## Materials and methods

**Figure 1.**
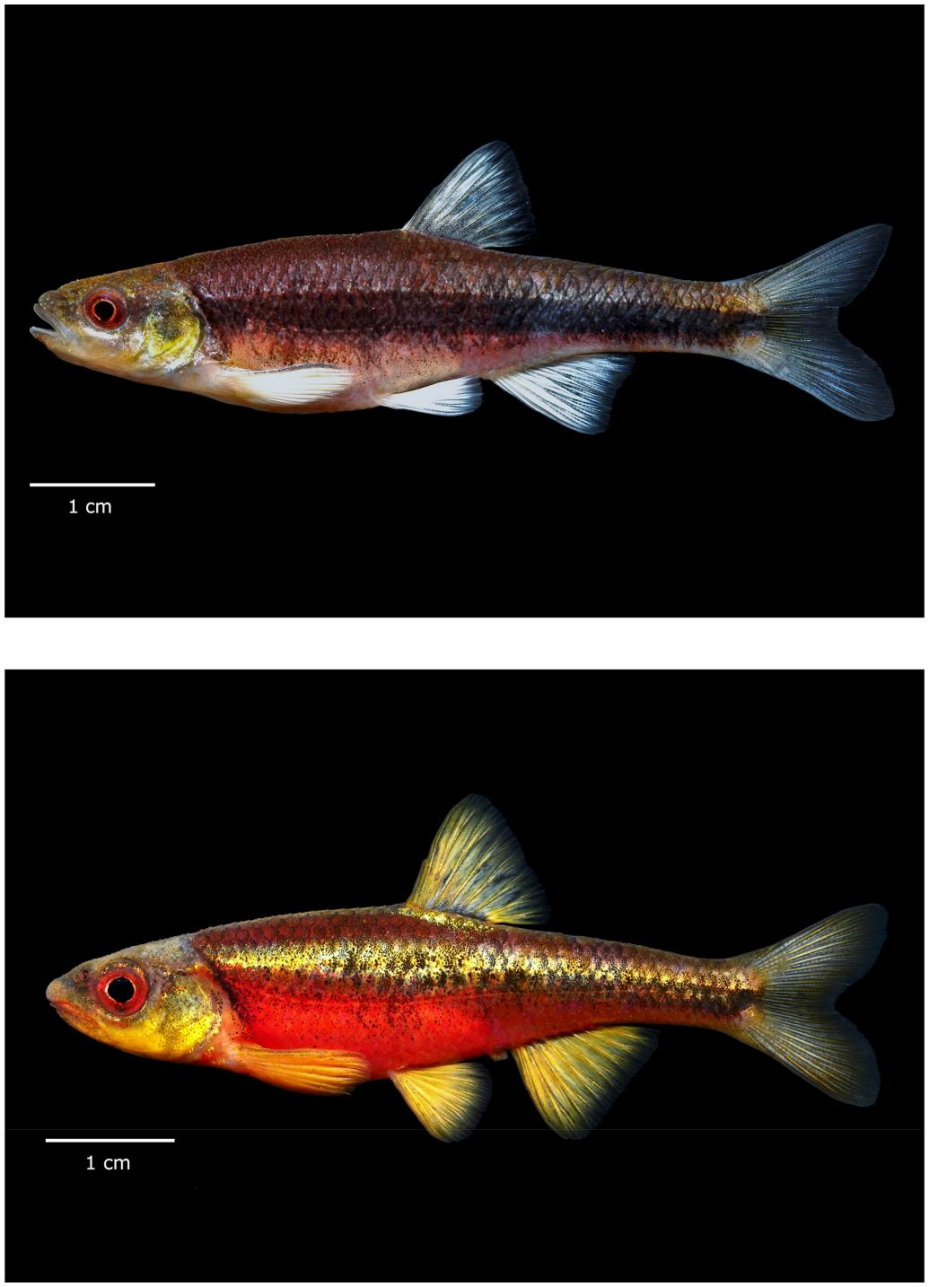
Representative photographs of (top) *Notropis chlorocephalus* (green-headed form) and (bottom) *N. chiliticus*. Photo credits: Zachariah D. Alley.

Specimens of the green-headed form of *N. chlorocephalus* were collected from Leepers Creek of the Catawba Watershed, North Carolina (35°29’55.7"N, 81°08’47.2"W). This species can readily be identified vs. congeners by their white fins, bright red body in nuptial adults, and green to yellow head (Figure 1). *Notropis chiliticus* was collected from Long Creek, Pee Dee basin, North Carolina (35°16’55.8"N, 80°14’52.9"W). *Notropis chiliticus* is characterized by red coloration on the lips, dorsal, anal, and caudal fins; breeding males also have a red body and yellow head and fins (Figure 1; Rohde et al. 2009). Pelvic fin clips were sub-sampled, and live specimens were released back to the site of capture. Fin clips were preserved in 95% ethanol and stored at −20 °C. Genomic DNA was extracted using the DNeasy Blood and Tissue Kit (QIAGEN) following instructions given by the manufacturer. DNA quality was assessed *via* electrophoresis using a 1.5% agarose gel. DNA concentration was determined with a NanoDrop 2000 Spectrophotometer (Thermo Fisher Scientific), then refrigerated at 4 °C. Specimens were deposited at Mississippi State University (Michael W. Sandel; mws297@msstate.edu) under voucher numbers ZA2108030301, ZA2108030302, and ZA2108030201.

The mitochondrial genome was amplified in two reactions using a GeneAmp PCR System 9700 (Applied Biosystems). Long-range PCR was conducted to amplify an ∼16,600bp segment. Reactions were performed at a total volume of 50 µl: PrimeSTAR GXL DNA Polymerase, 1X PrimeSTAR GXL Buffer (TaKaRa), 200 μM each dNTP, 0.2 µM primers (Table 1), 100 ng DNA, and UltraPure Water (Invitrogen) to volume. Cycling parameters included 30 cycles: 98 °C for 10 sec and 68 °C for 10 min followed by a final hold of 4 °C. The remaining segment of the genome (∼600bp) was amplified in a 25 µl reaction using PuReTaq Ready-To-Go^™^ PCR Beads (Cytiva) following manufacturer’s instructions, 0.4 µM primers (Table 1), 4 µM human blocking primer, and UltraPure Water to volume. Thermal cycling included an initial denaturation of 95 °C for 2 min; 35 cycles of 94 °C for 30 sec, 56 °C for 45 sec, and 72 °C for 1 min; a final extension of 72 °C for 10 min, and hold at 4 °C. Amplification was confirmed *via* gel electrophoresis (Figure S1) and products purified using ExoSAP-IT PCR Product Cleanup Reagent (Applied Biosystems; Bell 2008).

**Table 1.** PCR primers used for amplification of the mitochondrial genome. Primers were designed using Primer3 v.4.1.0 (Koressaar and Remm 2007; Untergasser et al. 2012; Kõressaar et al. 2018) on targets selected from alignments of vertebrate sequences.

Primer name	Primer sequence 5’→3’
**Long-range PCR**	
Vmt16s30L	ACGATTAAAGTCCTACGTGATCTGAGTTCA
Vmt16s30H	GATGTCCTGATCCAACATCGAGGTCGTAAA
**Standard PCR**	
16sar-LL	CGCCTGTTTACCAAAAACATCGCCTC
16sH3056S	CTCCGGTCTGAACTCAGATCACGTAG
16sar-Lblk (human-blocking)	CACCTCTAGCATCACCAGTATTAGAGGCACCG

PCR Sequencing was performed by Plasmidsaurus using Oxford Nanopore Technology. Reads were assembled in Geneious Prime v.2024.0.7 (Geneious 2025) to reference genome *Notropis lutipinnis* (MT333789) using the Geneious mapper under default parameters including Medium/Fast sensitivity and iterative fine-tuning (Figure S2). A consensus sequence was generated utilizing a 60% threshold. MitoAnnotator v.4.04 (Iwasaki et al. 2013; Sato et al. 2018) was used for annotation and Proksee Map Builder v2.0.5 (Grant et al. 2023) to produce genome maps.

All available mitochondrial genomes were collected for members of the genus *Notropis* and aligned in MAFFT v.7 (Katoh et al. 2002; Katoh and Standley 2013). A phylogenetic tree was reconstructed in CIPRES Science Gateway (Miller et al. 2010) using IQ-TREE v2.3.2 (Minh et al. 2020) under a Maximum Likelihood framework and ultrafast bootstraps (Hoang et al. 2018; 1,000 replicates). ModelFinder (Kalyaanamoorthy et al. 2017) was used to determine the appropriate substitution model (GTR+F + I + G4) and the tree edited in Figtree v1.4.4 (Rambaut 2007).

## Results

**Figure 2.**
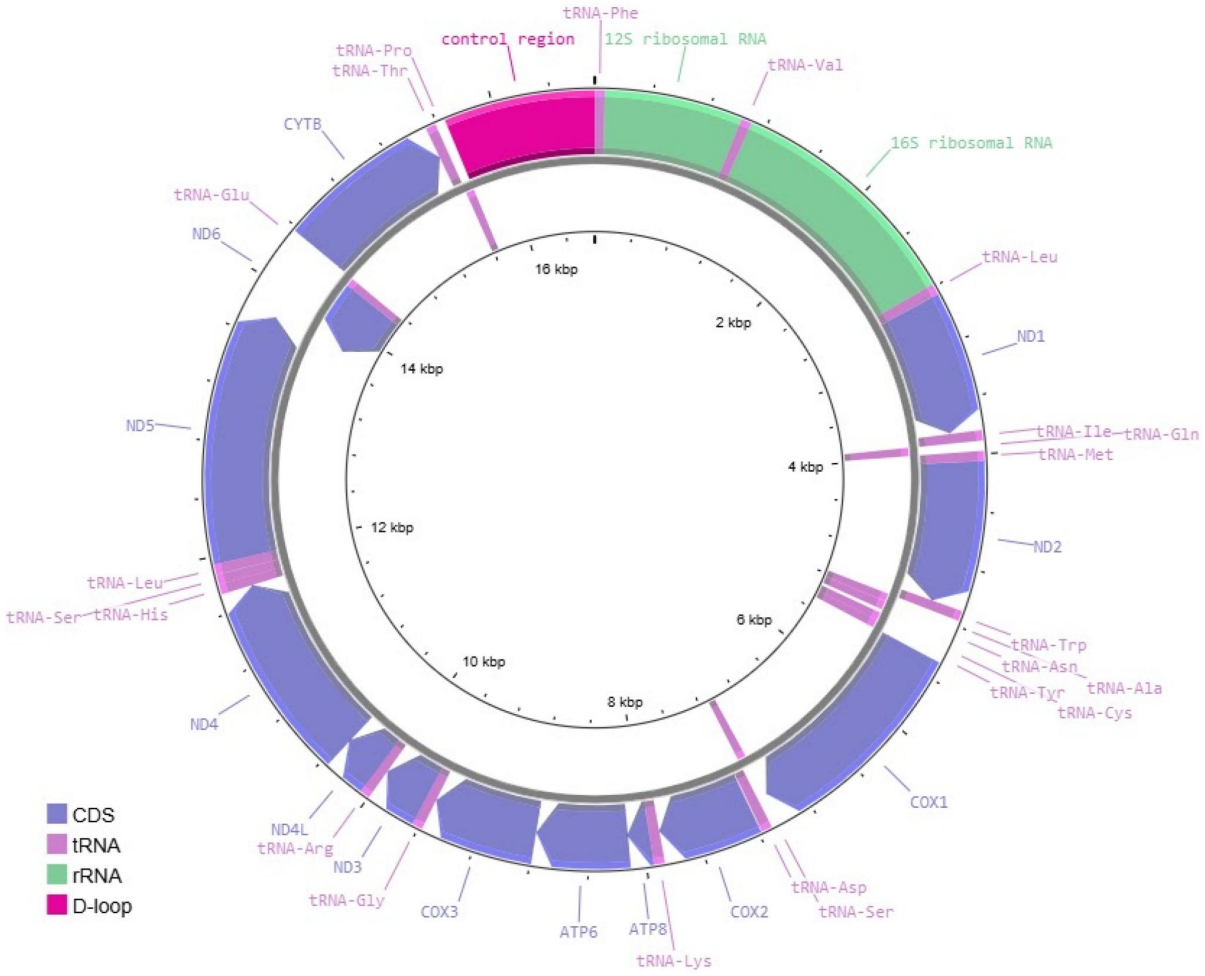
Mitochondrial genome map representing the highly similar gene orientation and content of *Notropis chiliticus* and *N. chlorocephalus.* The legend depicts using colors the gene type; CDS: coding sequence, tRNA: transfer RNA, rRNA: ribosomal RNA, D-loop: control region. Arrowheads indicate gene orientation.

The genomes of *N. chlorocephalus* contained 16,706bp and *N. chiliticus* 16,711bp, comprising a total of 13 protein coding genes, 22 transfer RNAs (tRNAs), two ribosomal RNAs (rRNAs), and a control region (D-loop; Figure 2). Length differences are in the control region, tRNA-Tyr, 16S rRNA, and an intergenic region. Nucleotide composition ranges consisted of 28.68–28.71% A, 26.25–26.85% C, 17.58–17.67% G, and 26.77–27.46% T. Percent identity between the two *N. chlorocephalus* genomes was 99.9% and 92.4% when compared to *N. chiliticus*.

## Discussion and conclusion

**Figure 3.**
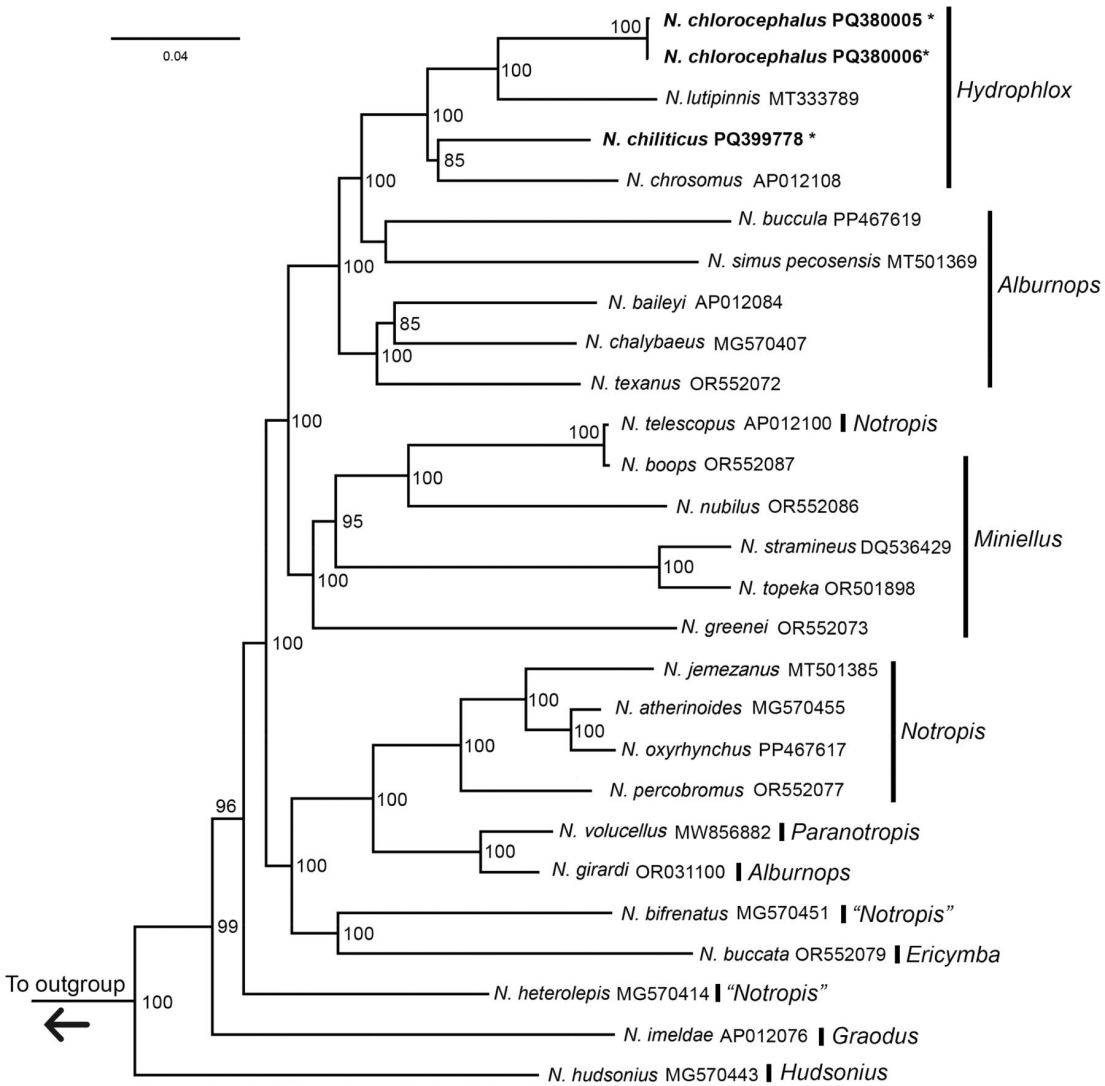
Maximum likelihood phylogeny constructed using mitochondrial genomes from the genus *Notropis*. Numbers on nodes are bootstrap support values; values below 70 are not shown. The sequences generated in this study are written in bold font and marked with asterisks. Taxonomic names on the right indicate contemporary suggested naming for groups within *Notropis* (Stout et al. 2022). The following sequences were used: PQ380005 (this study), PQ380006 (this study), MT333789 (Bobier 2020), PQ399778 (this study), AP012108, PP467619 (Fields et al. 2024), MT501369, AP012084 (Iwasaki et al. 2013), MG570407 (Schroeter et al. 2020), OR552072, AP012100, OR552087, OR552086, DQ536429 (Broughton and Reneau 2006), OR501898, OR552073, MT501385, MG570455 (Schroeter et al. 2020), PP467617 (Fields et al. 2024), OR552077, MW856882, OR031100, MG570451 (Schroeter et al. 2020), OR552079, MG570414 (Schroeter et al. 2020), AP012076, and MG570443 (Schroeter et al. 2020). The outgroup, *Chrosomus tennesseensis* (MZ097372; Wood et al. 2022), has been removed from the figure for clarity.

The mitochondrial genomes of *N. chlorocephalus* and *N. chiliticus* show high pairwise similarity and synteny. Congruent with previous phylogenomic analyses, we recovered the *Hydrophlox* group (including *N. chlorocephalus* and *N. chiliticus*) as monophyletic (Figure 3; Cashner et al. 2011; Schönhuth et al. 2018; Bobier 2020; Stout et al. 2022). Though phylogenies based on morphological and behavioral traits resolved *Notropis baileyi* as a member of the *Hydrophlox* clade (Swift 1970), our analysis supports its inclusion in the *Alburnops* clade as have prior molecular studies (Cashner et al. 2011; Schönhuth et al. 2018; Bobier 2020; Stout et al. 2022). The groups *Alburnops*, *Miniellus*, and *Notropis* were not recovered as monophyletic clades, and the tree topology is not congruent with other phylogenetic studies (Schönhuth et al. 2018; Stout et al. 2022).

Discrepancies in tree topology between data types generated in prior studies could be attributed to the differing evolutionary histories of mitochondrial and nuclear genomes (Forsythe et al. 2021). The addition of other members of *Hydrophlox* (e.g. *N. rubricroceus*), as well as other members of the shiner clade, could aid in our understanding of the evolutionary relationships within the group. Availability of these genomes will assist investigators in developing eDNA primers, support contemporary inferences demonstrating relationships within *Notropis* and will benefit investigators in need of sequences for investigations focused on North American Leuciscids.

## Supplementary Material

Revision_Alley 2024 Notropis Mito B Supplemental.docx

## Data Availability

Data supporting the findings of this study are available on the open-source platform Genbank of NCBI, and can be found at (https://www.ncbi.nlm.nih.gov/) (PQ380005, PQ380006, and PQ399778). The BioProject number is PRJNA742674; the SRA numbers are SRR31000593, SRR31000594, and SRR31000592; and the BioSample numbers are SAMN44293012, SAMN44293013, and SAMN44293014. Raw sequence data are additionally available through the Scholars Junction open access digital repository (https://doi.org/10.54718/IOIW6793).
