# Supplementary material for: The complete mitochondrial genomes of Notropis chlorocephalus and Notropis chiliticus
Source: Mitochondrial DNA B Resour. 2025 Jan 23;10(2):139–43. doi: 10.1080/23802359.2025.2457449 (PMC11758796; doi:10.1080/23802359.2025.2457449)
Supplement: Revision_Alley 2024 Notropis Mito B Supplemental.docx [file TMDN_A_2457449_SM0534.docx]

Supplementary Material

The Complete Mitochondrial Genomes of *Notropis chlorocephalus* and *Notropis chiliticus*

Zachariah D. Alley^1^, Kayla M. Fast^2^, & Michael W. Sandel^2,3^

1-Protected Species Unit, EDGE Engineering and Science, Houston, TX, 77084, USA

2-Department of Wildlife, Fisheries and Aquaculture, Mississippi State University, Mississippi State, MS, 39762, USA

3-Forest and Wildlife Research Center, Mississippi State University, Mississippi State, MS, 39762, USA

Correspondence: Kayla M. Fast Department of Wildlife, Fisheries and Aquaculture, Mississippi State University, Mississippi State, MS, USA and Michael W. Sandel Department of Wildlife, Fisheries and Aquaculture, Mississippi State University, Mississippi State, MS, USA. Email: kmf160@msstate.edu (KMF) and mws297@msstate.edu (MWS)

ORCID

Zachariah D. Alley: 0000-0002-8448-6583

Kayla M. Fast: 0000-0001-5476-5330

Michael W. Sandel: 0000-0001-9083-9202

Journal Name

Mitochondrial DNA Part B


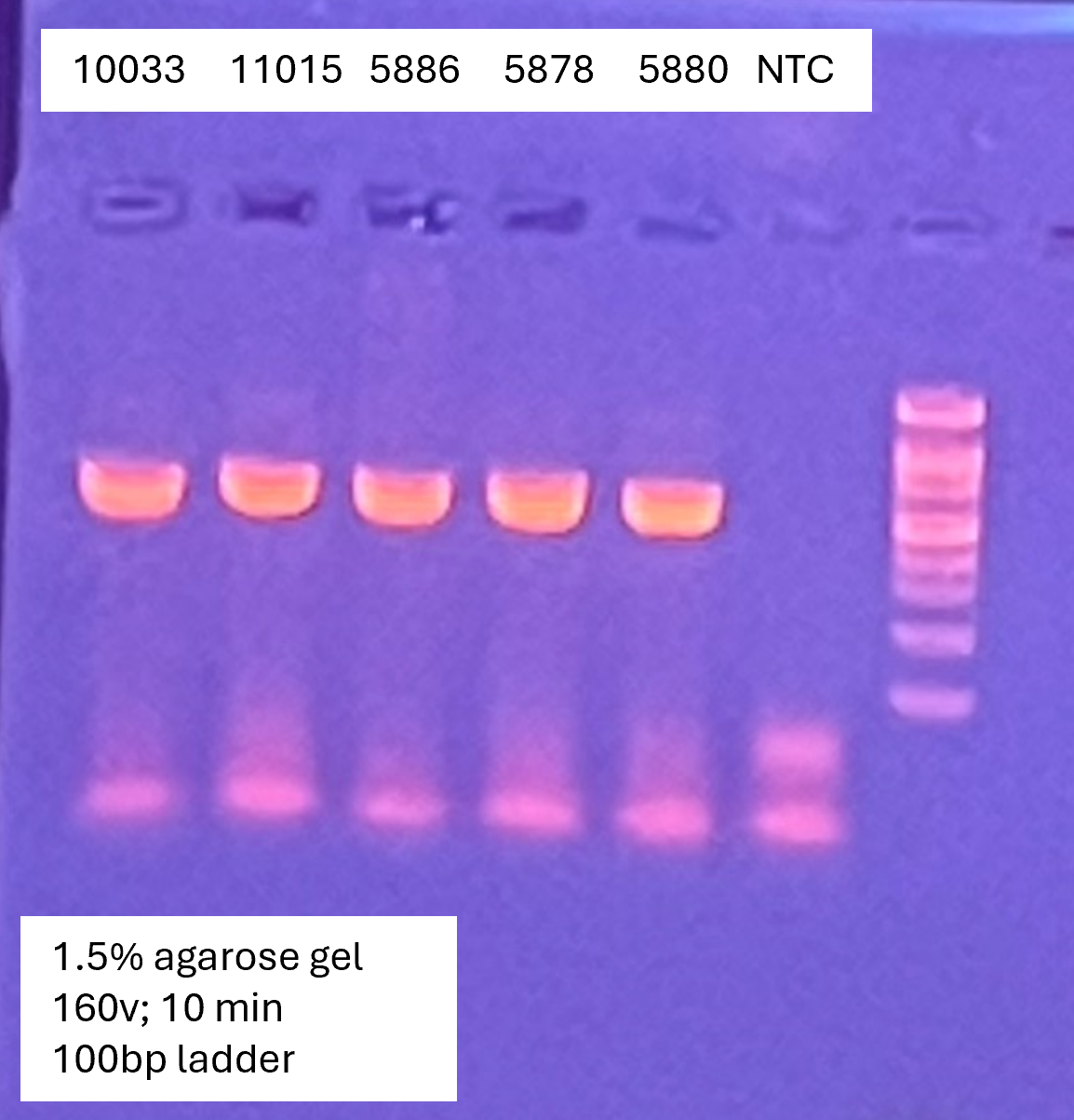

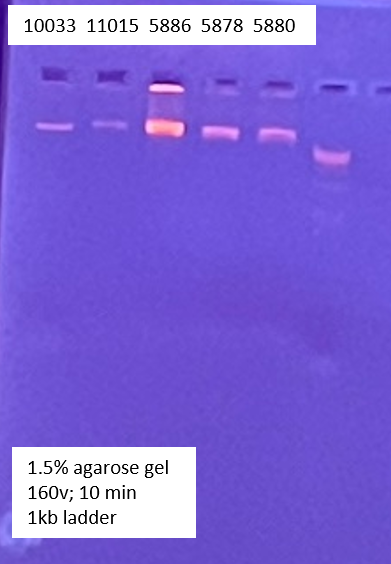


Figure S1. Gel electrophoresis results after PCR amplification. Left: Standard PCR product (~600 bp); right: long-amp PCR product (~16,600 bp). *Notropis chlorocephalus* (5878-PQ380006 and 5880-PQ380005) and *N. chiliticus* (5886-PQ399778).


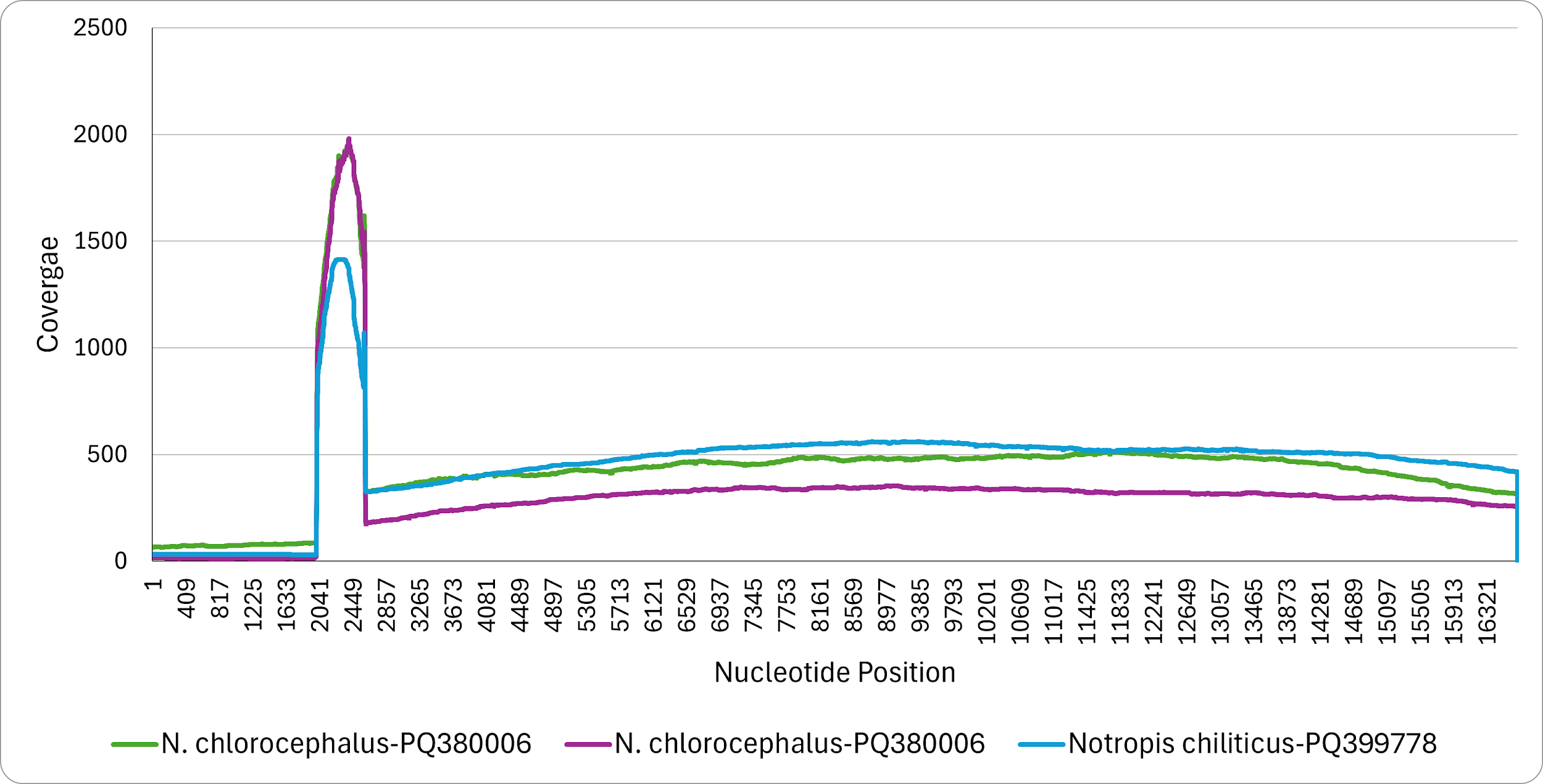


Figure S2. Depth of read coverage from mitochondrial genome sequencing.
